# Scenarios for the long‐term efficacy of amyloid‐targeting therapies in the context of the natural history of Alzheimer's disease

**DOI:** 10.1002/alz.14134

**Published:** 2024-07-29

**Authors:** Lars Lau Raket, Jeffrey Cummings, Alexis Moscoso, Nicolas Villain, Michael Schöll

**Affiliations:** ^1^ Eli Lilly and Company Indianapolis Indiana USA; ^2^ Clinical Memory Research Unit Department of Clinical Sciences Lund University Lund Sweden; ^3^ Chambers‐Grundy Center for Transformative Neuroscience Pam Quirk Brain Health and Biomarker Laboratory Department of Brain Health School of Integrated Health Sciences University of Nevada Las Vegas (UNLV) Las Vegas Nevada USA; ^4^ Wallenberg Centre for Molecular and Translational Medicine and the Department of Psychiatry and Neurochemistry University of Gothenburg Huvudbyggnad Vasaparken, Universitetsplatsen 1 Gothenburg Sweden; ^5^ Department of Neurology Institute of Memory and Alzheimer's Disease AP‐HP Sorbonne Université Pitié‐Salpêtrière Hospital Paris France; ^6^ Sorbonne Université INSERM U1127 Institut du Cerveau ‐ ICM Paris France; ^7^ Department of Clinical Physiology Sahlgrenska University Hospital Gothenburg Sweden; ^8^ Dementia Research Centre Queen Square Institute of Neurology University College London London UK

**Keywords:** amyloid‐targeting therapies, clinical trials, disease modeling, disease modification, long‐term efficacy, time saving

## Abstract

**INTRODUCTION:**

Recent clinical trials of amyloid beta (Aβ)‐targeting therapies in Alzheimer's disease (AD) have demonstrated a clinical benefit over 18 months, but their long‐term impact on disease trajectory is not yet understood. We propose a framework for evaluating realistic long‐term scenarios.

**METHODS:**

Results from recent phase 3 trials of Aβ‐targeting antibodies were integrated with an estimate of the long‐term patient‐level natural history trajectory of the Clinical Dementia Rating‐Sum of Boxes (CDR‐SB) score to explore realistic long‐term efficacy scenarios.

**RESULTS:**

Three distinct long‐term efficacy scenarios were examined, ranging from conservative to optimistic. These extrapolations of positive phase 3 trials suggested treatments delayed onset of severe dementia by 0.3 to 0.6 years (conservative), 1.1 to 1.9 years (intermediate), and 2.0 to 4.2 years (optimistic).

**DISCUSSION:**

Our study provides a common language for long‐term impact of disease‐modifying treatments. Our work calls for studies with longer follow‐up and results from early intervention trials to provide a comprehensive assessment of these therapies' true long‐term impact.

**Highlights:**

- We present long‐term scenarios of the efficacy of AD therapies.
- In this framework, scenarios are defined relative to the natural history of AD.
- Long‐term projections with different levels of optimism can be compared.
- It provides a common language for expressing beliefs about long‐term efficacy.

## 1 BACKGROUND

Alzheimer's disease (AD) is a progressive neurodegenerative disease characterized by the pathological accumulation of amyloid beta (Aβ) and tau aggregates in the brain.^1^ Although the exact mechanism of AD pathogenesis remains unclear, current neuropathologic, genetic, and human in vivo studies strongly support the *amyloid cascade hypothesis*,^2^ which posits Aβ as a key agent in the pathologic process of AD. Aβ has thus been among the most common therapeutic targets of experimental drugs for patients with AD.^3^

After years of failed attempts to develop clinically effective Aβ‐targeting therapies, converging evidence indicates that therapies targeting Aβ aggregates that reduce brain Aβ plaque load below ∼20 to 25 Centiloids on Aβ positron emission tomography (PET), that is, the threshold commonly used to define Aβ positivity, has clinical benefits for patients with early symptomatic AD.^4^ This is evidenced by statistically significant reductions of 22% to 36% in decline in the Clinical Dementia Rating‐Sum of Boxes (CDR‐SB) score across the EMERGE (aducanumab),^5^ CLARITY AD (lecanemab),^6^ and TRAILBLAZER‐ALZ 2 (donanemab)^7^ trials, with associated amyloid clearance rates of 48%, 68%, and 80%. The ENGAGE (aducanumab) and GRADUATE I and II (gantenerumab) confirmatory trials did show significant amyloid reductions with amyloid clearance rates of 31%, 28%, and 27%,^5^, ^8^ but they failed to show statistically significant clinical benefits with observed reductions in decline of −2%, 8%, and 6%. However, an exploratory post hoc analysis in the GRADUATE I and II trials of gantenerumab suggested that these negative outcomes likely reflected the failure to achieve the desired degree of Aβ plaque removal.^8^

While these results represent a milestone in the search for effective disease‐modifying therapies, the clinical and economic relevance of the observed treatment effects, particularly beyond the 18‐month trial duration, remain a subject of debate and carry implications for decisions about the potential implementation and reimbursement of these treatments.^9^, ^10^, ^11^, ^12^

To better understand the significance of the novel Aβ‐targeting therapies, it is crucial to understand treatment effects in the context of the natural history of AD. Abnormal Aβ accumulation is a slow process that begins years before the onset of symptoms,^13^ as reflected by the fact that many cognitively unimpaired older individuals (∼30% over 70 years of age) have elevated Aβ plaque loads in their brain (and are thus defined as Aβ‐positive).^14^ While this phase of being cognitively unimpaired with Aβ‐positive status can last many years, it is associated with a greatly increased risk of progressing to symptomatic AD over the long term.^15^, ^16^ Most trials of Aβ‐targeting therapies, including the three positive confirmatory trials to date, have primarily focused on Aβ‐positive subjects with early symptomatic AD. Intervening in this population increases the likelihood of demonstrating a clinical benefit over a shorter time span compared to trials with Aβ‐positive cognitively unimpaired individuals. There is a hypothesis that addressing Aβ pathology before symptoms could delay or entirely prevent cognitive decline due to AD.^17^ This hypothesis has yet to be confirmed for the Aβ‐targeting monoclonal antibodies that have demonstrated efficacy in the early symptomatic stages of AD, but trials with longer‐term follow‐up in presymptomatic individuals are under way.^18^, ^19^

**RESEARCH IN CONTEXT**

1. **Systematic review**: The authors reviewed both the primary literature on trial results of aducanumab, lecanemab, donanemab, and gantenerumab and published commentary. This review revealed diverging views on the efficacy and implications of these trials, reflecting ongoing debates in the field regarding the interpretation of amyloid beta (Aβ)‐targeting therapies' effects on Alzheimer's disease (AD) progression.
2. **Interpretation**: This study aimed to integrate disparate perspectives on Aβ‐targeting therapies by developing a common language for discussing potential long‐term efficacy relative to the natural history of AD.
3. **Future directions**: Future research should address the uncertainties surrounding the true long‐term impact of Aβ‐targeting therapies. This calls for studies with longer‐term follow‐up, early intervention trials, and examinations of real‐world effectiveness.

This article addresses scenarios for long‐term treatment effects of recent Aβ‐targeting therapies. To address the question in a framework that is consistent with AD progression, we used an estimate of the typical patient‐level natural history trajectory of CDR‐SB from the earliest stages of preclinical AD to severe AD dementia. The estimated trajectory was based on data from the Alzheimer's Disease Neuroimaging Initiative (ADNI) using a previously validated disease progression model.^20^ This long‐term reference trajectory allowed for the exploration of different scenarios for long‐term treatment effects. We used published data from the recent trials of high‐potency Aβ‐targeting therapies to model long‐term treatment effects relative to the natural history trajectory of CDR‐SB. The results of our study contribute to elucidating the potential of Aβ‐targeting therapies to produce and maintain clinically meaningful benefits over varying time durations.

## 2 METHODS

### 2.1 Clinical studies and efficacy results

This study focused on data from positive phase 3 trials of high‐clearance Aβ‐targeting antibodies: EMERGE of aducanumab, CLARITY AD of lecanemab, and the primary population (low/medium tau) in TRAILBLAZER‐ALZ 2 of donanemab. For completeness, the Supplementary Material includes analyses of data from ENGAGE, EMERGE and ENGAGE combined, GRADUATE I and II, and the high‐tau and combined low/medium and high tau populations of TRAILBLAZER‐ALZ 2. The trials included in this analysis had slightly different entry criteria. For example, the range of allowable Mini‐Mental Status Examination (MMSE) scores at baseline differed among studies. All the trials involved early AD with overlapping population characteristics.

The EMERGE and ENGAGE studies included both a high and a low dose of aducanumab; however, since the high dose is recommended,^21^ the low dose was not considered here. The primary patient population in TRAILBLAZER‐ALZ 2 had a low/medium tau load based on [^18^F]flortaucipir PET retention patterns and quantification (standardized uptake value ratios [SUVR]). The study also included a population of patients with high tau load but excluded patients with no or very low tau load. We focus on the results of the primary low/medium tau population but report results from the high tau and combined low/medium and high tau populations in the Supplementary Material.

Baseline scores and trajectories of change in CDR‐SB and associated standard deviations were extracted from publications or public presentations. For comparability, we used trajectories estimated using categorical time models. Most studies reported results based on the mixed model for repeated measures (MMRM), but results for the GRADUATE I and II studies were reported based on a reference‐based multiple imputation analysis of covariance (ANCOVA) model that is closely related to MMRM. All results were reported as estimated marginal means representing an average patient in the trial. For all studies, the CDR‐SB results at the final visits were reported, but some intermediate data points required extraction from graphs using WebPlotDigitizer.^22^

The time delay at the final visit and associated time saving (time delay divided by trial duration) were estimated from the extracted MMRM trajectories using natural cubic spline interpolation of the placebo results and computing the time at which the placebo trajectory crossed the score of the active‐arm at the final visit.

### 2.2 Natural history trajectory of CDR‐SB

A long‐term patient‐level natural history trajectory of CDR‐SB from the earliest stages of preclinical AD to late‐stage dementia was estimated based on a previously published disease progression model.^20^ The detailed specification of the model and estimated trajectory have been described.^23^ Briefly, the model is a latent‐time disease progression model that simultaneously estimated trajectories of CDR‐SB, ADAS‐cog (13‐item), MMSE, and Aβ PET (Centiloids) based on longitudinal observations (4581 follow‐up years) from 1424 well‐characterized subjects from ADNI. Subjects were either Aβ negative and cognitively unimpaired or Aβ positive with any cognitive status. Based on the continuous‐time staging of patients, the natural history CDR‐SB trajectory was estimated based on all available observations of CDR‐SB using a mixed‐effects model with the mean trajectory modeled as a natural cubic spline model with 5 degrees of freedom and a patient‐level random intercept. The time scale of the natural history trajectory is the time since Aβ PET positivity, which means that 0 corresponds to the time when the estimated mean curve for Aβ PET crosses the positivity threshold of 24.1 Centiloids. The estimated CDR‐SB trajectory used for this study is specified in Table S1 in the Supplementary Material.

We explored the effects of intervening at different disease stages with hypothetical interventions that resulted in an enduring delay, cumulative stage‐dependent (fading) slowing of disease progression, and stage‐independent slowing of disease progression. Based on placebo trajectories, we predicted the time to severe dementia defined as a CDR‐SB score of 16 and the predicted treatment‐associated time delays compared to placebo in progressing to severe dementia.

### 2.3 Integrating clinical trial trajectories with natural history trajectories

We used average baseline CDR‐SB scores in the clinical studies to identify the starting points of the trial trajectories along the natural history trajectory of CDR‐SB.

The active‐arm and placebo‐arm trajectories were extrapolated throughout the disease course. For the placebo arms, a post‐trial decline proportional to the natural history trajectory was assumed (horizontal shift of natural history trajectory to match the placebo arm at the final visit), while for the active arms, three different assumptions were explored:

1. **Enduring short‐term delay**. The time delay of progression relative to placebo observed at the end of the double‐blind treatment exposure was enduring, but after that, disease continued to follow the natural history trajectory. For example, if a 6‐month delay in disease progression was observed at the end of the trial, then all subsequent milestones occurred with a 6‐month delay relative to the extrapolated placebo trajectory.
2. **Stage‐dependent fading slowing**. The time saving observed at the end of the trial (time delay divided by trial duration) was assumed to continue at a decaying rate past the end of the trial. The rate of time saving was assumed to decrease linearly from the observed slowing at the end of the trial to 0% time saving once the trajectory reached CDR‐SB = 16, at which point the trajectory continued to follow the natural history trajectory. For example, if a 6‐month delay in disease progression was observed at the end of an 18‐month trial (33%), and it took 72 months from baseline for the extrapolated active treatment arm trajectory to reach CDR‐SB = 16, the delays at 36, 54, and 72 months after treatment initiation would be 11 months (31%), 14 months (26%), and 15 months (21%), respectively. After 72 months, no additional increase in the delay would occur (15 months delay until reaching CDR‐SB = 18).
3. **Stage‐independent continued slowing**. The time saving observed at the end of the trial (time delay divided by trial duration) was assumed to continue at the same rate past the end of the trial, where patients followed the natural history trajectory at a slower pace. For example, if a 6‐month delay in disease progression was observed at the end of an 18‐month trial (33%), disease progression extrapolated by the natural history trajectory would be delayed by 12 months 36 months after treatment initiation (33%).

The first scenario was considered the most conservative disease‐modifying scenario, where no additional benefit accrued past the end of the trial period, while the third scenario was considered optimistic. The second scenario is an intermediate scenario between scenarios 1 and 2.

There are two main sources of uncertainty associated with the long‐term extrapolation procedure described above. The first has to do with the chosen scenario and the second with the treatment effect extracted from the trial. The first type of uncertainty can be addressed by considering different scenarios, and the second can be addressed by considering long‐term extrapolations with a range of treatment effects around the one extracted. Due to the extraction of time‐saving estimates from summary‐level data, the uncertainty of the estimate cannot be accurately assessed to establish, for example, standard errors. However, time‐saving estimates computed on the basis of patient‐level data with associated uncertainty quantification are reported from the TRAILBLAZER‐ALZ 2 trial.^7^ In this trial, a standard error of approximately 5% points was reported for percentage time‐saving estimates for CDR‐SB in both the low/medium tau and combined low/medium and high‐tau populations. To assess the impact of uncertainty in the extracted time saving, we considered the impact of increasing or decreasing the estimate by 10% points, which, under a setting similar to that of the TRAILBLAZER‐ALZ 2 study, approximately corresponds to boundaries of the 95% confidence interval of the percentage time saving.

## 3 RESULTS

**FIGURE 1:**
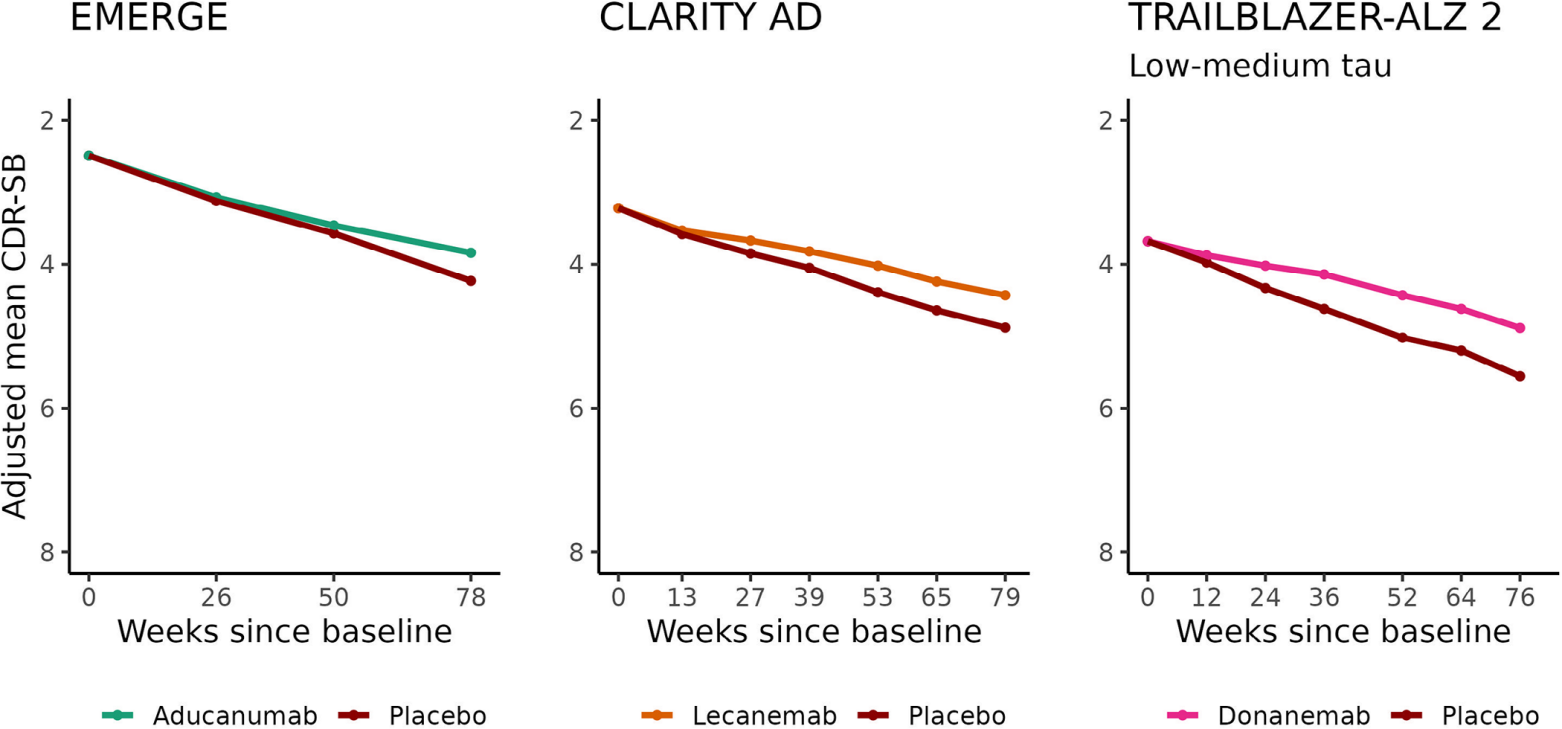
Clinical Dementia Rating‐Sum of Boxes (CDR‐SB) results of EMERGE, CLARITY AD, and TRAILBLAZER‐ALZ 2. Results are based on publicly reported results of change from baseline in CDR‐SB analyzed using the mixed model for repeated measures with the reported average baseline score added to the results to bring them to the CDR‐SB scale.

The adjusted mean CDR‐SB trajectories for EMERGE, CLARITY AD, and TRAILBLAZER‐ALZ 2 are shown in Figure 1.

The characteristics of the trials, disease severity of the trial populations at baseline, and quantifications of treatment effects (treatment differences, time delays) are shown in Table 1. The disease severity of the trial populations differed in terms of their mean baseline CDR‐SB scores and predicted years since Aβ PET positivity of the average participants. The results suggest that the population in EMERGE was the least progressed, followed by CLARITY AD, and then TRAILBLAZER‐ALZ 2. The characteristics and estimated treatment effects of all trials and subpopulations are given in Table S2 in the Supplementary Material.

**TABLE 1:** Characteristics of trials and estimated treatment effects on CDR‐SB at final visit.

Trial	Treatment	Trial duration (weeks)	Mean baseline CDR‐SB	Predicted time since Aβ PET positivity at baseline (years)	CDR‐SB difference at final visit	Estimated time delay at final visit (weeks)	Estimated time saving at final visit (%)
EMERGE	Aducanumab	78	2.49	10.0	−0.39	15.7	20
CLARITY AD	Lecanemab	79	3.22	10.7	−0.45	24.3	31
TRAILBLAZER‐ALZ 2	Donanemab	76	3.68	11.1	−0.67	30.1	40

**FIGURE 2:**
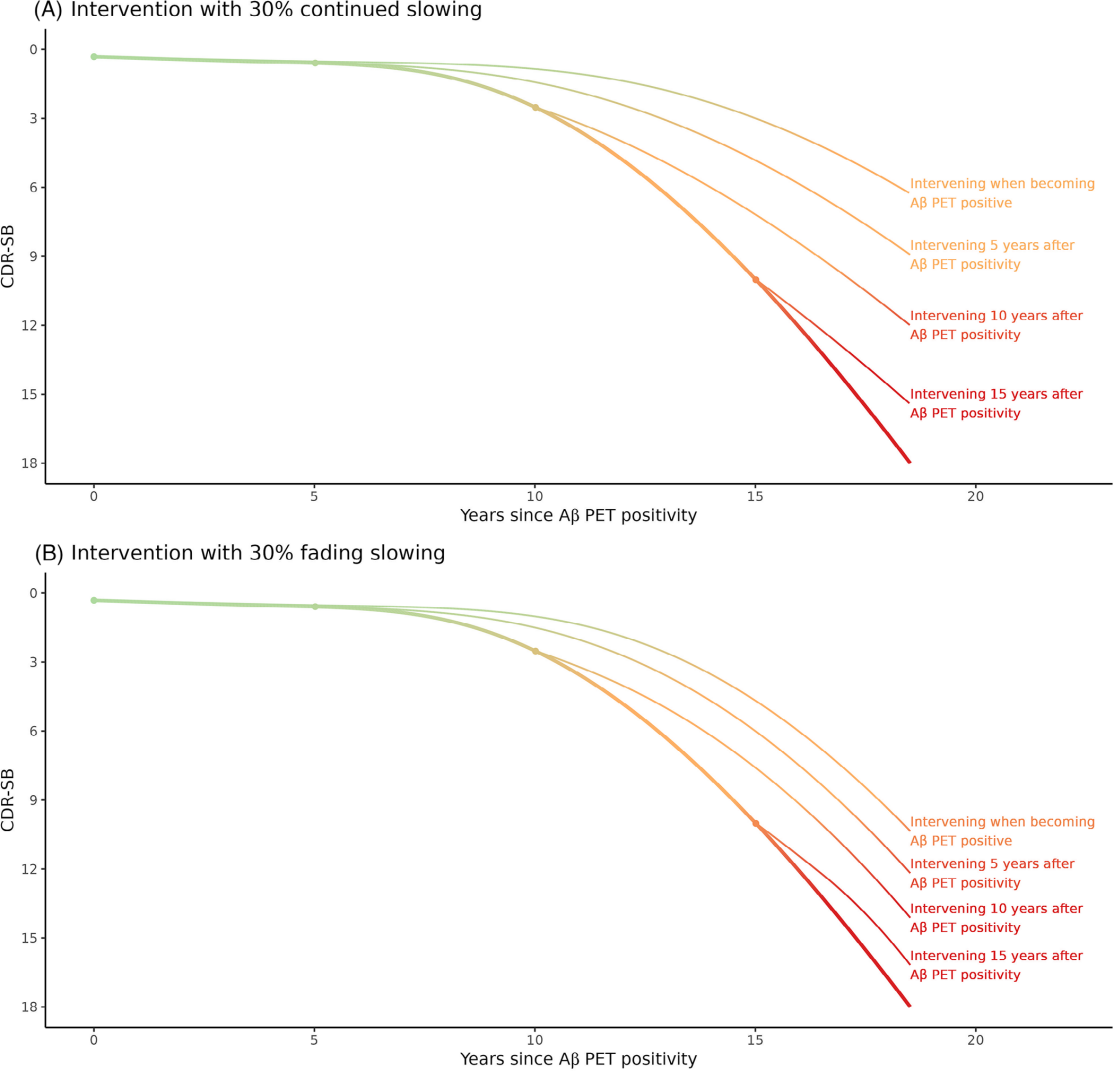
Estimated natural history trajectory of Clinical Dementia Rating‐Sum of Boxes (thick line) from Raket et al.^23^ Four examples (thin lines) illustrate how hypothetical interventions that produce (A) 30% stage‐independent continued slowing of disease progression and (B) 30% stage‐dependent fading slowing would change the trajectory based on when the intervention was started (0, 5, 10, or 15 years after Aβ PET positivity).

The estimated natural history trajectory of CDR‐SB is shown in Figure 2, along with different treatment effect scenarios. Figure 2A illustrates the effect of intervening at different time points with a treatment that results in 30% continued stage‐independent slowing, and Figure 2B illustrates the effect of intervening at the same time points with a treatment that results in 30% fading stage‐dependent slowing. This illustrates the long‐term benefit of intervening earlier in disease progression if the treatment effect accumulates over time. Severe dementia, defined as a CDR‐SB score of at least 16, occurred 17.7 years after Aβ PET positivity. In the 30% continued slowing scenario, when intervening at the time of becoming Aβ PET positive, severe dementia was delayed by 7.6 years. When intervening 5, 10, and 15 years after amyloid positivity, severe dementia was delayed by 5.4, 3.3, and 1.2 years, respectively. In the 30% fading slowing scenario, the corresponding delays were 3.4, 2.5, 1.6, and 0.7 years.

**FIGURE 3:**
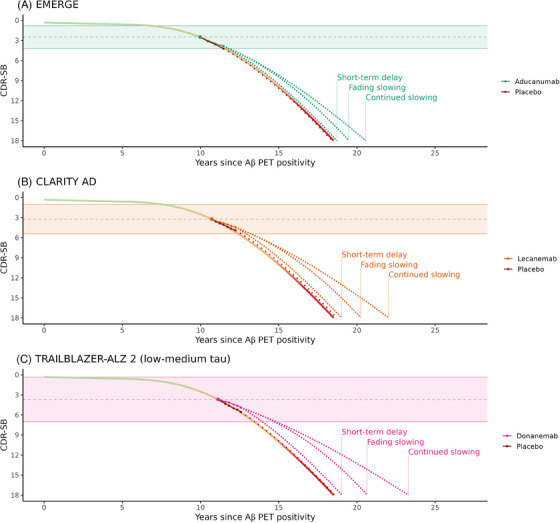
Trial results mapped to long‐term Clinical Dementia Rating‐Sum of Boxes (CDR‐SB) trajectory with extrapolations (dotted lines). The gradient trajectory represents the estimated natural history trajectory, and the shaded bands represent the 90% prediction intervals for baseline CDR‐SB scores in the different studies.

For each of the positive trials, the three long‐term efficacy scenarios are shown in Figure 3. Despite the differences in baseline disease stage across trials, the benefits associated with long‐term trajectories were consistent with trial results, with TRAILBLAZER‐ALZ 2 (most progressed patients) showing the best long‐term results, followed by CLARITY AD and EMERGE (least progressed). Consistent with the results, the GRADUATE I and II trials and ENGAGE showed the least long‐term benefit in all scenarios (Figures S1 and S2). To illustrate the effect of uncertainty in the extracted estimates of percentage time saving, the continued slowing scenario is illustrated in Figure S3, along with an uncertainty band computed by percentage time saving that is 10% less or greater than the extracted estimate.

The projected CDR‐SB differences from placebo and associated time delays of disease progression at 3, 4, and 5 years after treatment initiation in the individual trials are given in Table S3 in the Supplementary Material. The estimated delays in time to severe dementia and associated uncertainty intervals are given in Table 2.

**TABLE 2:** Extrapolated estimates of time to severe dementia (CDR‐SB = 16).

			Treatment‐associated delay in years to severe dementia		
Trial	Treatment	Years to severe dementia (placebo)	Short‐term delay	Fading slowing	Continued slowing
EMERGE	Aducanumab	7.6	0.3 [0.2, 0.5]	1.1 [0.6, 1.7]	2.0 [1.0, 3.3]
CLARITY AD	Lecanemab	7.2	0.5 [0.3, 0.6]	1.4 [0.8, 2.0]	2.9 [1.7, 4.5]
TRAILBLAZER‐ALZ 2	Donanemab	6.6	0.6 [0.4, 0.7]	1.9 [1.3, 2.6]	4.2 [2.7, 6.2]

*Note*: Uncertainty estimates are represented by numbers in brackets, computed by decreasing and increasing the extracted percentage time‐saving estimate by 10% points.

Comparisons of EMERGE and ENGAGE and a combined analysis are shown in Figures S4 and S5 in the Supplementary Material. Comparisons of the primary low/medium tau population, the high tau population, and the combined low/medium and high tau population in TRAILBLAZER‐ALZ 2 are shown in Figures S6 and S7 in the Supplementary Material.

## 4 DISCUSSION

This study explored different long‐term efficacy scenarios of Aβ‐targeting therapies in the context of the natural history trajectory of AD. The findings shed light on some of the challenges and complexities of evaluating and comparing the clinical efficacy of Aβ‐targeting therapies based on the currently available trial results. The modeling presented here highlights the importance of comparing trial results in a manner that considers both differences in trial populations and trial duration, which will influence the observed treatment differences due to the highly non‐linear natural history trajectory of CDR‐SB over the course of AD.

Among our three long‐term scenarios, one was considered conservative and one optimistic. The conservative scenario assumed that the benefit accrued during the trial period was lasting, but that the disease would progress according to the natural history trajectory with no additional benefit after the trial period. The optimistic scenario extrapolated the time saving observed during the study period to the remaining disease course. In the optimistic scenario, patients kept declining, but the reason for considering this optimistic is that we found it unlikely that amyloid clearance could result in substantially increasing benefit after the end of the trial periods in symptomatic AD patients, given the existence of pathological tau and copathologies such as vascular disease, Lewy bodies, and TAR DNA‐binding protein 43  aggregates in the vast majority of these patients.^24^ While it is conceivable that the full benefit of Aβ‐targeting therapies was not observed within the study periods for all subjects (eg, subjects not reaching clearance or reaching clearance at the final visit), it is our hypothesis that amyloid clearance in the early symptomatic stages of AD is likely to result in long‐term trajectories within the spans of the conservative and optimistic long‐term trajectories shown in Figure 3. Our scenario with fading stage‐dependent slowing is one example of an intermediate scenario, but one could construct infinitely many different scenarios within this span.

The rationale for a continued slowing of disease progression beyond the clinical trial duration is primarily rooted in the observation that amyloid pathology facilitates the spreading of tau pathology^25^ and, thus, acts as an accelerant of clinical disease progression. However, due to the “prion‐like” nature of pathological tau,^26^ the time saving related to Aβ‐targeting therapies could diminish in later‐stage disease where more widespread tau pathology may be less reliant on amyloid‐related pathways to accelerate spreading and propagation.^25^, ^27^, ^28^ Similarly, cell death and accrual of non‐Aβ, non‐tau pathologies may reflect the shape of the disease trajectory in later years. These observations motivated our scenario with a stage‐dependent fading slowing. An assumption that may be challenged is that the slowing observed in the individual trial was an inherent result of the given therapy in the chosen trial population. While the different Aβ‐targeting monoclonal antibodies differ in epitopes,^29^ which may result in unique properties,^30^ an alternative assumption could be that the observed efficacy is fully driven by the speed and depth of amyloid clearance. This assumption would suggest that the greater time saving seen in the TRAILBLAZER‐ALZ 2 trial was driven by the observed faster and deeper amyloid clearance rather than donanemab's specific target. The implication would be that once subjects reach a sufficiently low global amyloid load, they will experience the full benefit of amyloid clearance that will be independent of the therapy used.

Understanding the long‐term impact of disease‐modifying therapies is of key importance across stakeholders, including patients, prescribers, regulators, and payers. However, analyses that project potential benefits into the future are primarily done in the setting of health economic studies. Typical health economic models use Markov models to describe coarse disease stage transitions (eg, from mild cognitive impairment [MCI] to mild dementia).^31^, ^32^, ^33^ These models differ from our proposed framework in two important aspects. First, Markov models focus on discrete stages, each associated with a burden that only changes when stage transitions occur. The discrete stages may directly reflect disease stage (eg, mild dementia) but can also include events that may or may not be directly related to disease progression (eg, moving to a care facility or death). Second, the models assume the Markov property, which means that the probability of progressing to another stage within a given timeframe only depends on the current stage but is independent of the history of progression, including how long the patient has been in the current state.

A Markov model for predicting lifetime health outcomes associated with aducanumab treatment suggested that median time delay to transition from MCI to severe AD dementia among those alive was 2.8 years,^34^ which is substantially greater than our most optimistic estimate of delay in the onset of severe dementia of 2.0 years, which was only based on the EMERGE trial results. A more elaborate health economic model based on conditional event simulation was used to simulate long‐term health outcomes of lecanemab based on the CLARITY AD trial.^35^ This analysis suggested that lecanemab could delay severe AD dementia by 2.2 years, which falls between our intermediate (1.4 years) and optimistic (2.9 years) scenarios.

This study focused on the continuous progression of disease and quantified treatment effects in terms of time delays and time saving to avoid some of the pitfalls of working with other quantifications of treatment effects, such as treatment differences and proportional reduction in decline. For example, proportional reduction in decline can be unstable when trajectories are non‐linear and lead to unnatural extrapolations (eg, patients on active treatment can never fully decline).^36^ However, our focus on time‐based treatment effects required us to compute treatment effects based on summary data. Time‐saving estimates from CLARITY AD based on a mixed‐effects model assuming a linear trajectory of patient‐level data have been reported.^37^ This patient‐level analysis estimated that lecanemab delayed progression by 5.3 months after 18 months of treatment. This estimate corresponds to a 29% time saving, which is very similar to the 31% extracted from the MMRM in the present analysis. In TRAILBLAZER‐ALZ 2, it was reported that donanemab resulted in a 33‐week delay of disease progression after 76 weeks of treatment, corresponding to a 43% time saving.^7^ These results were based on a proportional time‐saving Progression Model for Repeated Measures (PMRM) analysis of patient‐level data.^36^ The time‐delay estimate based on the MMRM trajectories that was used for the present analysis was similar with an estimated 40% time saving.

In addition to the limitations of the long‐term extrapolation scenarios mentioned previously, the study also had limitations in terms of the anchoring of studies on the estimated CDR‐SB natural history trajectory. The different studies had different inclusion criteria, which might have led to biased sampling of subjects along the disease timeline, causing the trial trajectories not to align with the estimated natural progression. In the studies analyzed, we found that the placebo groups’ progression patterns generally matched the estimated natural history well, except for the high tau population in TRAILBLAZER‐ALZ 2 (Figure S5). It seems likely that the inclusion of patients that are required to have symptoms consistent with early symptomatic disease while also having a high tau load biased this population to have a greater proportion of patients with more advanced disease (consistent with high tau load) than their symptom profile at screening revealed. This suggests that the primary low/medium tau population of TRAILBLAZER‐ALZ 2 is the most representative population for comparison because it excluded no/very low tau subjects and high tau subjects. This is corroborated by the fact that the placebo group closely matched the natural history trajectory. These observations point to a general limitation: the analyses in the present study were based on matching adjusted mean trajectories from trials to a trajectory that was estimated to represent a typical patient with AD. Caution is warranted when interpreting these results in relation to subgroups or individual patient‐level trajectories, where factors such as AD pathological load, copathologies, and cognitive reserve may influence the rate of decline and, potentially, the response to treatment.^38^, ^39^, ^40^

The focus of this study was the efficacy of Aβ‐targeting therapies, but we did not address differences in safety profiles. The primary adverse events of interest for the class of Aβ‐targeting therapies are amyloid‐related imaging abnormalities (ARIA). Differences in ARIA rates have been observed across phase 3 trials but should not be directly compared due to differences in trial populations that affected the baseline risk of ARIA, including differences in AD pathological load, comorbidities, and concomitant medications. To date, the TRAILBLAZER‐ALZ 4 trial is the only head‐to‐head trial available that compared two Aβ‐targeting therapies, namely, donanemab and aducanumab.^41^ The results of this trial illustrate that across therapies, ARIA rates are to a greater degree a function of the therapeutic molecule than the speed of amyloid clearance. To better understand safety differences across Aβ‐targeting therapies, more studies in comparable populations are needed.

In summary, the Aβ‐targeting treatments that have shown significant impact on clinical outcomes over 18 months delayed disease progression on CDR‐SB between 4 and 7 months during the study periods. Based on the long‐term scenarios examined in this study, this may lead to delays of severe dementia by 4 to 7 months (conservative), 1.1 to 1.9 years (intermediate), or 2.0 to 4.2 years (optimistic) if treatment is initiated in the early symptomatic stages of AD. If amyloid clearance results in cumulative benefits like the continued and fading slowing scenarios, the most substantial long‐term benefits would occur if Aβ‐targeting treatments were used in the presymptomatic stage of AD.

## CONFLICT OF INTEREST STATEMENT

LLR is an employee and shareholder of Eli Lilly and Company. JC has provided consultation to Acadia, Actinogen, Acumen, AlphaCognition, Aprinoia, AriBio, Artery, Biogen, BioVie, Cassava, Cerecin, Diadem, EIP Pharma, Eisai, GemVax, Genentech, GAP Innovations, Janssen, Jocasta, Karuna, Lilly, Lundbeck, LSP, Merck, NervGen, Novo Nordisk, Oligomerix, Optoceutics, Ono, Otsuka, PRODEO, Prothena, ReMYND, Roche, Sage Therapeutics, Signant Health, Simcere, Suven, SynapseBio, TrueBinding, Vaxxinity, and Wren pharmaceutical, assessment, and investment companies. AM reports no conflicts of interest. NV has received research support from Fondation Bettencourt‐Schueller, Fondation Servier, Union Nationale pour les Intárêts de la Mádecine (UNIM), Fondation Claude Pompidou, Fondation Alzheimer and Fondation pour la Recherche sur l'Alzheimer; travel grants from the Movement Disorders Society, Merz‐Pharma, UCB Pharma, and GE Healthcare SAS; is an unpaid local principal investigator or subinvestigator in NCT04241068 and NCT05310071 (aducanumab, Biogen), NCT05399888 (BIIB080, Biogen), NCT03352557 (gosuranemab, Biogen), NCT05463731 (remternetug, Eli Lilly), NCT04592341 (gantenerumab, Roche), NCT03887455 (lecanemab, Eisai), NCT03828747 and NCT03289143 (semorinemab, Roche), NCT04619420 (JNJ‐63733657, Janssen—Johnson & Johnson), NCT04374136 (AL001, Alector), NCT04592874 (AL002, Alector), NCT04867616 (bepranemab, UCB Pharma), NCT04777396 and NCT04777409 (semaglutide, Novo Nordisk), NCT05469360 (NIO752, Novartis), is an unpaid national coordinator for NCT05564169 (masitinib, ABScience), NCT (AD04, ADvantage Therapeutics GmbH); has given unpaid lectures at symposia organized by Eisai and the Servier Foundation; and has been an unpaid expert for Janssen and Johnson & Johnson, all outside of this work. MS has served on advisory boards for and received funding from Roche Diagnostics and Novo Nordisk (outside scope of submitted work). Author disclosures are available in the supporting information.

## CONSENT STATEMENT

Not applicable.

## Supporting information

Supporting Information

Supporting Information
