# Supplementary material for: Scenarios for the long‐term efficacy of amyloid‐targeting therapies in the context of the natural history of Alzheimer's disease
Source: Alzheimers Dement. 2024 Jul 29;20(9):6374–83. doi: 10.1002/alz.14134 (PMC11497713; doi:10.1002/alz.14134)
Supplement: Supplementary file 1 — Supporting Information [file ALZ-20-6374-s001.docx]

Scenarios for the long-term efficacy of amyloid-targeting therapies in the context of the natural history of Alzheimer’s disease

Lars Lau Raket, Jeffrey Cummings, Alexis Moscoso, Nicolas Villain, Michael Schöll

Supplementary Material

**Table S1.** Estimated values of natural history CDR-SB trajectory from 0 to 18.5 years since Aβ PET positivity.

| \| Years since Aβ PET positivity \| CDR-SB \| \| --- \| --- \| \| 0.0 \| 0.31 \| \| 0.5 \| 0.34 \| \| 1.0 \| 0.37 \| \| 1.5 \| 0.41 \| \| 2.0 \| 0.44 \| \| 2.5 \| 0.46 \| \| 3.0 \| 0.49 \| \| 3.5 \| 0.52 \| \| 4.0 \| 0.54 \| \| 4.5 \| 0.56 \| \| 5.0 \| 0.59 \| \| 5.5 \| 0.62 \| \| 6.0 \| 0.67 \| \| 6.5 \| 0.75 \| \| 7.0 \| 0.85 \| \| 7.5 \| 0.99 \| \| 8.0 \| 1.18 \| \| 8.5 \| 1.42 \| \| 9.0 \| 1.72 \| | \| Years since Aβ PET positivity \| CDR-SB \| \| --- \| --- \| \| 9.5 \| 2.09 \| \| 10.0 \| 2.51 \| \| 10.5 \| 2.99 \| \| 11.0 \| 3.54 \| \| 11.5 \| 4.15 \| \| 12.0 \| 4.82 \| \| 12.5 \| 5.55 \| \| 13.0 \| 6.34 \| \| 13.5 \| 7.18 \| \| 14.0 \| 8.07 \| \| 14.5 \| 9.01 \| \| 15.0 \| 10.00 \| \| 15.5 \| 11.03 \| \| 16.0 \| 12.10 \| \| 16.5 \| 13.20 \| \| 17.0 \| 14.35 \| \| 17.5 \| 15.52 \| \| 18.0 \| 16.73 \| \| 18.5 \| 17.97 \| |
| --- | --- | --- | --- | --- | --- | --- | --- | --- | --- | --- | --- | --- | --- | --- | --- | --- | --- | --- | --- | --- | --- | --- | --- | --- | --- | --- | --- | --- | --- | --- | --- | --- | --- | --- | --- | --- | --- | --- | --- | --- | --- | --- | --- | --- | --- | --- | --- | --- | --- | --- | --- | --- | --- | --- | --- | --- | --- | --- | --- | --- | --- | --- | --- | --- | --- | --- | --- | --- | --- | --- | --- | --- | --- | --- | --- | --- | --- | --- | --- | --- | --- |

**Table S2.** Characteristics of trial populations and estimated treatment effects on CDR-SB at final visit.

| Trial | Treatment | Trial duration | Mean baseline CDR-SB | Predicted time since Aβ PET positivity at baseline | CDR-SB difference at final visit | Estimated time delay at final visit | Estimated time saving at final visit |
| --- | --- | --- | --- | --- | --- | --- | --- |
| EMERGE | Aducanumab | 78 weeks | 2.49 | 10.0 years | -0.39 | 15.7 weeks | 20% |
| ENGAGE | Aducanumab | 78 weeks | 2.40 | 9.9 years | 0.03 | −1.1 weeks | −1% |
| CLARITY AD | Lecanemab | 79 weeks | 3.22 | 10.7 years | -0.45 | 24.3 weeks | 31% |
| TRAILBLAZER-ALZ 2  Low-medium tau | Donanemab | 76 weeks | 3.68 | 11.1 years | -0.67 | 30.1 weeks | 40% |
| TRAILBLAZER-ALZ 2  High tau | Donanemab | 76 weeks | 4.40 | 11.7 years | -0.69 | 15.5 weeks | 20% |
| TRAILBLAZER-ALZ 2  Low-medium and high tau | Donanemab | 76 weeks | 3.90 | 11.3 years | -0.70 | 22.3 weeks | 29% |
| GRADUATE I | Gantenerumab | 116 weeks | 3.71 | 11.1 years | -0.31 | 6.1 weeks | 5% |
| GRADUATE II | Gantenerumab | 116 weeks | 3.52 | 11.0 years | -0.19 | 11.1 weeks | 10% |

**
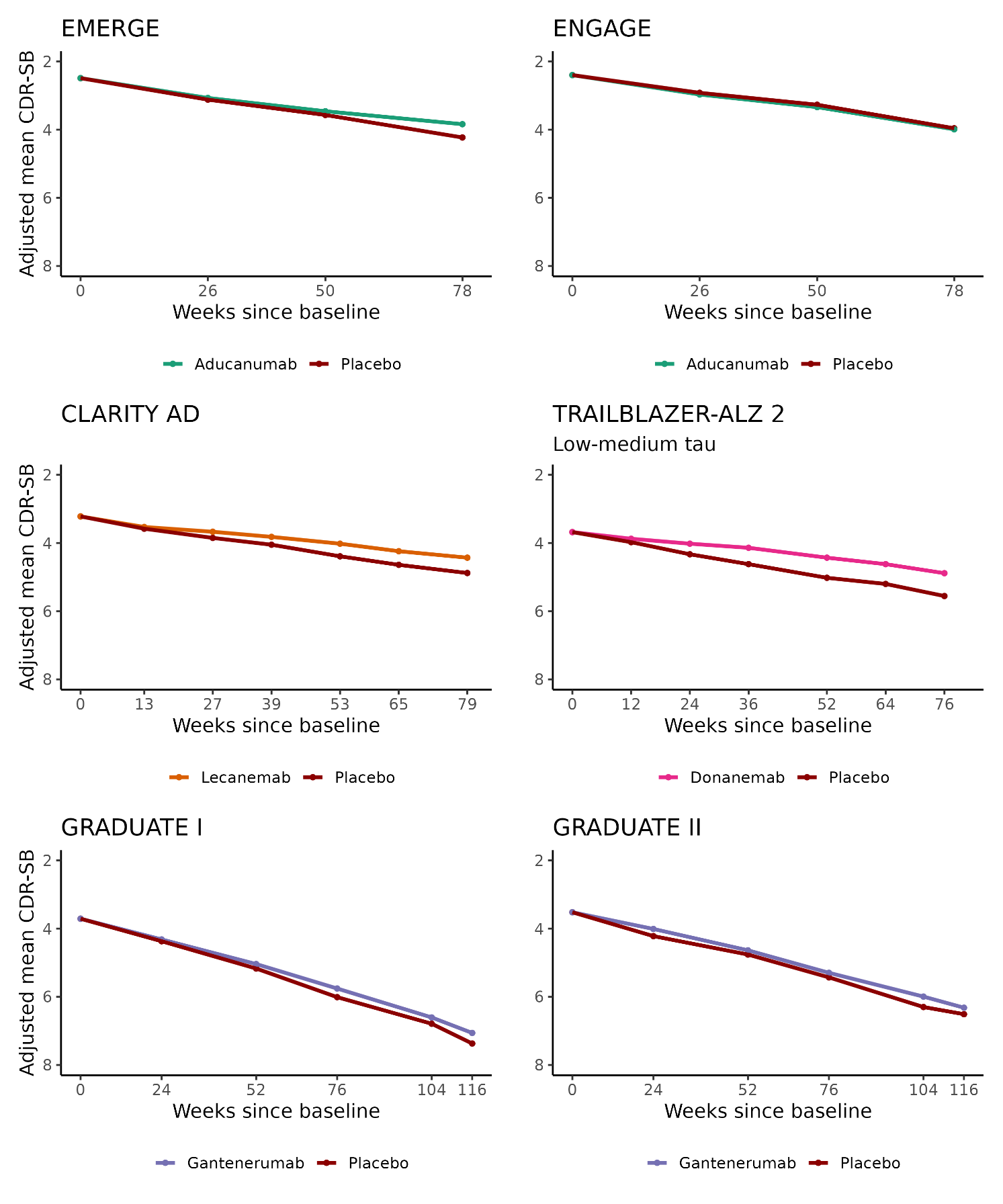
Figure S1**. Clinical Dementia Rating sum of boxes (CDR-SB) results of trials and treatment groups included in the present study. Results are based on publicly reported results of change from baseline in CDR-SB analyzed using the mixed model for repeated measures with the reported average baseline score added to the results to bring them to the CDR-SB scale


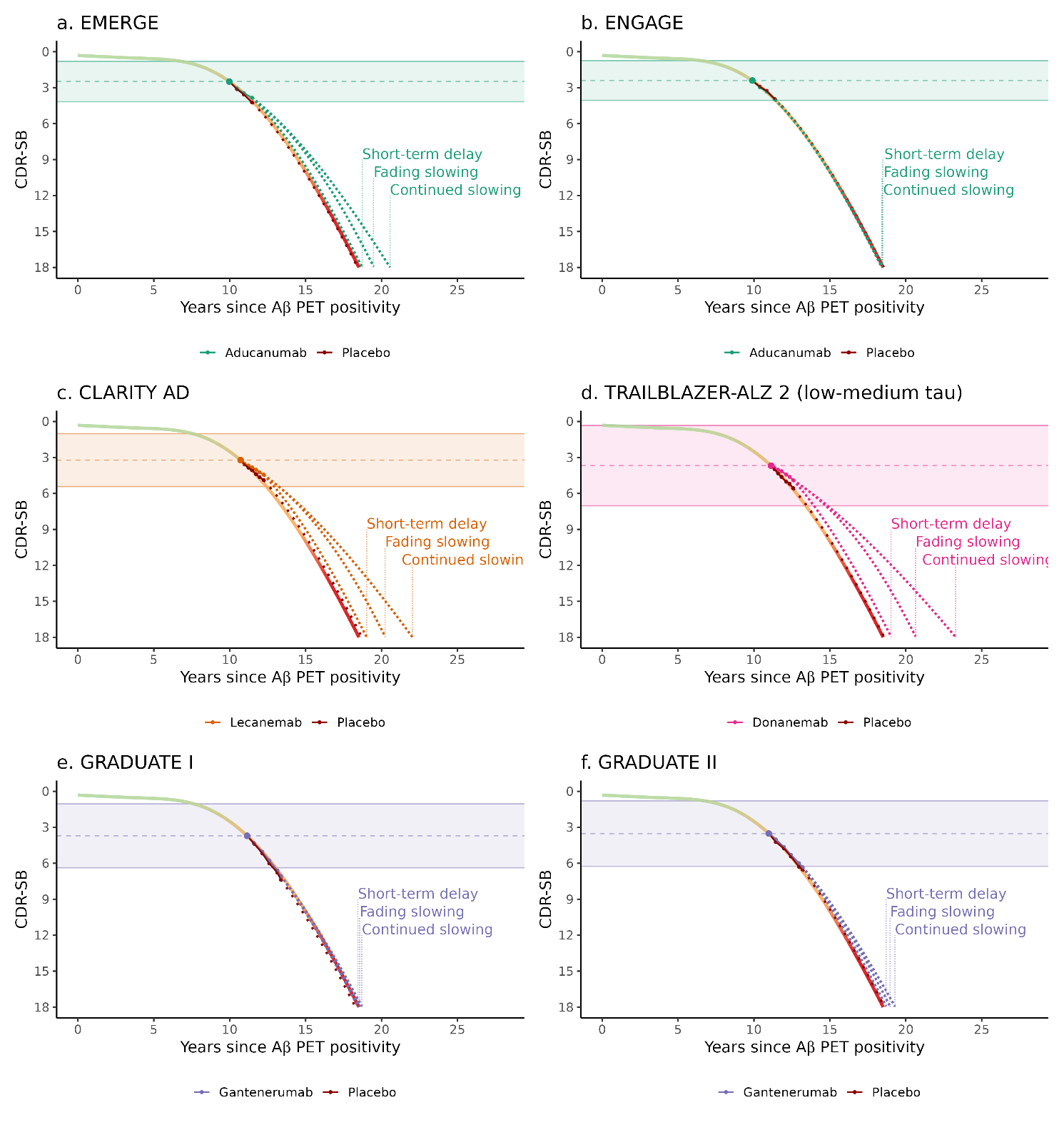


**Figure S2.** Trial results mapped to the long-term CDR-SB trajectory with extrapolations (dotted lines). The gradient trajectory represents the estimated natural history trajectory, and the shaded bands represent the 90% prediction intervals for baseline CDR-SB scores in the different studies.


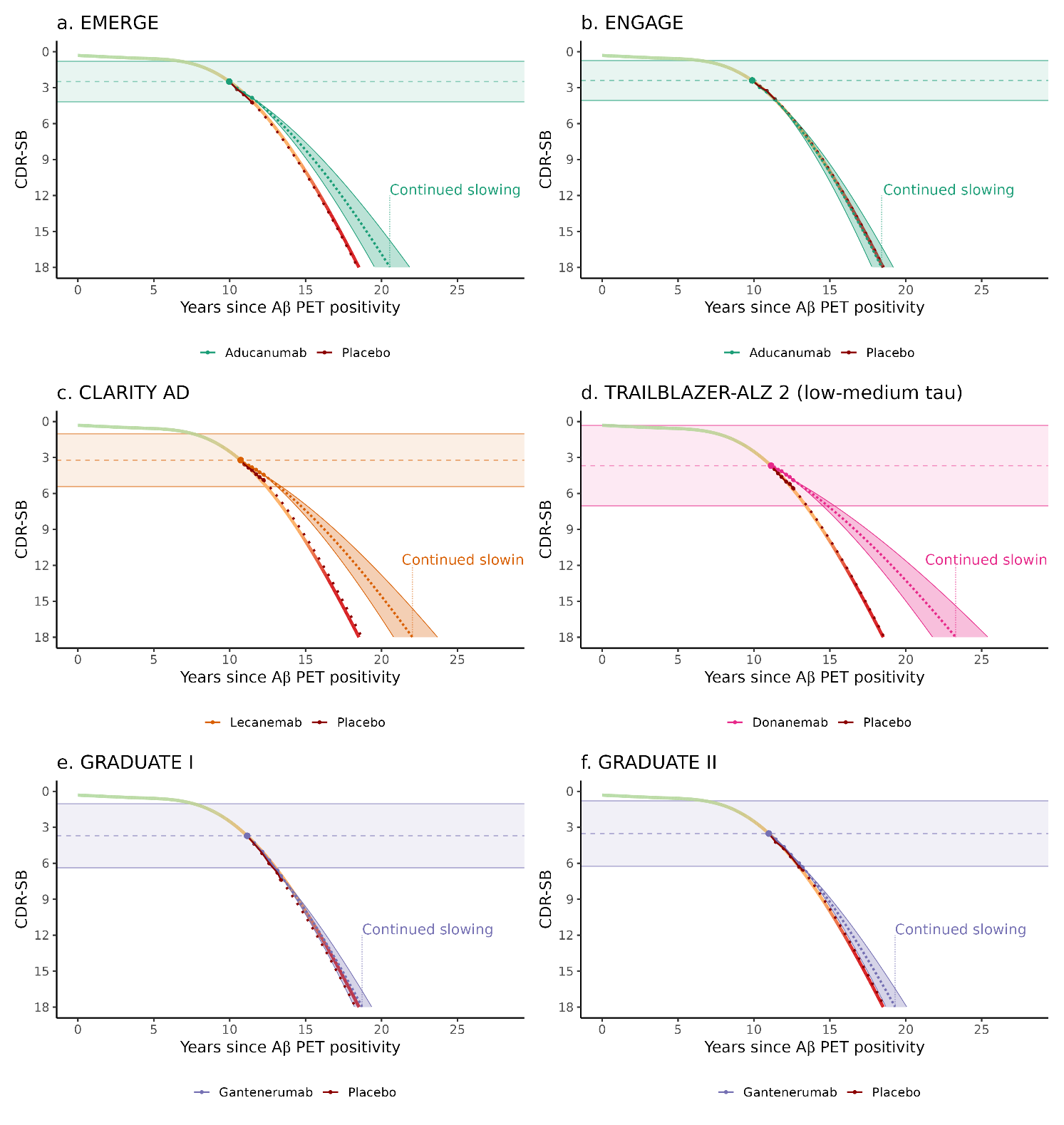


**Figure S3.** Trial results mapped to the long-term CDR-SB trajectory with continued slowing extrapolations (dotted lines) and uncertainty intervals (shaded areas around extrapolation) representing the extrapolation with a percent time saving estimate that is 10% points lower and higher than the extracted estimates. The gradient trajectory represents the estimated natural history trajectory, and the shaded horizontal bands represent the 90% prediction intervals for baseline CDR-SB scores in the different studies.


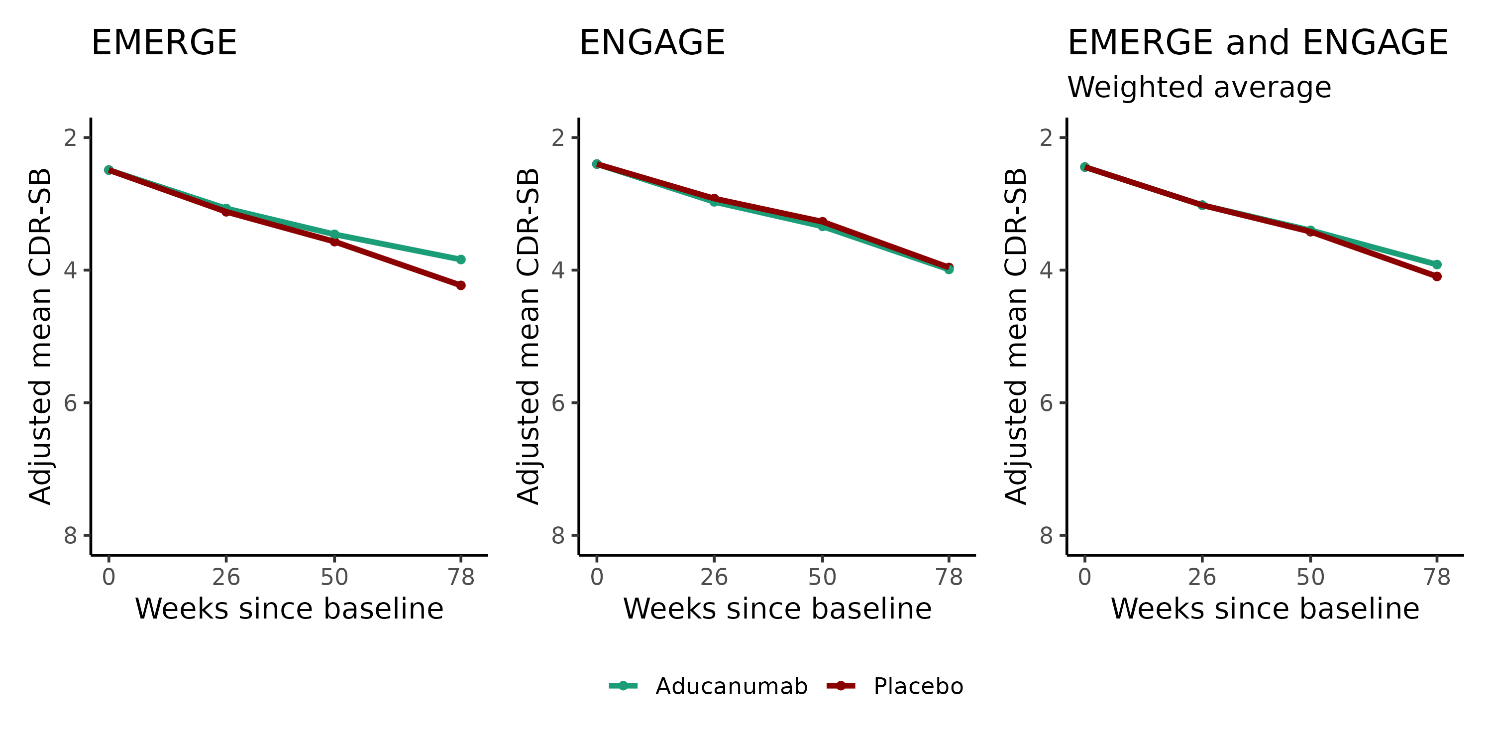


**Figure S4**. Clinical Dementia Rating sum of boxes (CDR-SB) results of the EMERGE and ENGAGE trials and a weighted average. Results are based on previously reported results of change from baseline in CDR-SB analyzed using the mixed model for repeated measures with the reported average baseline score added to the results to bring them to the CDR-SB scale. The weighted average is weighted by the baseline allocation to treatment arms.


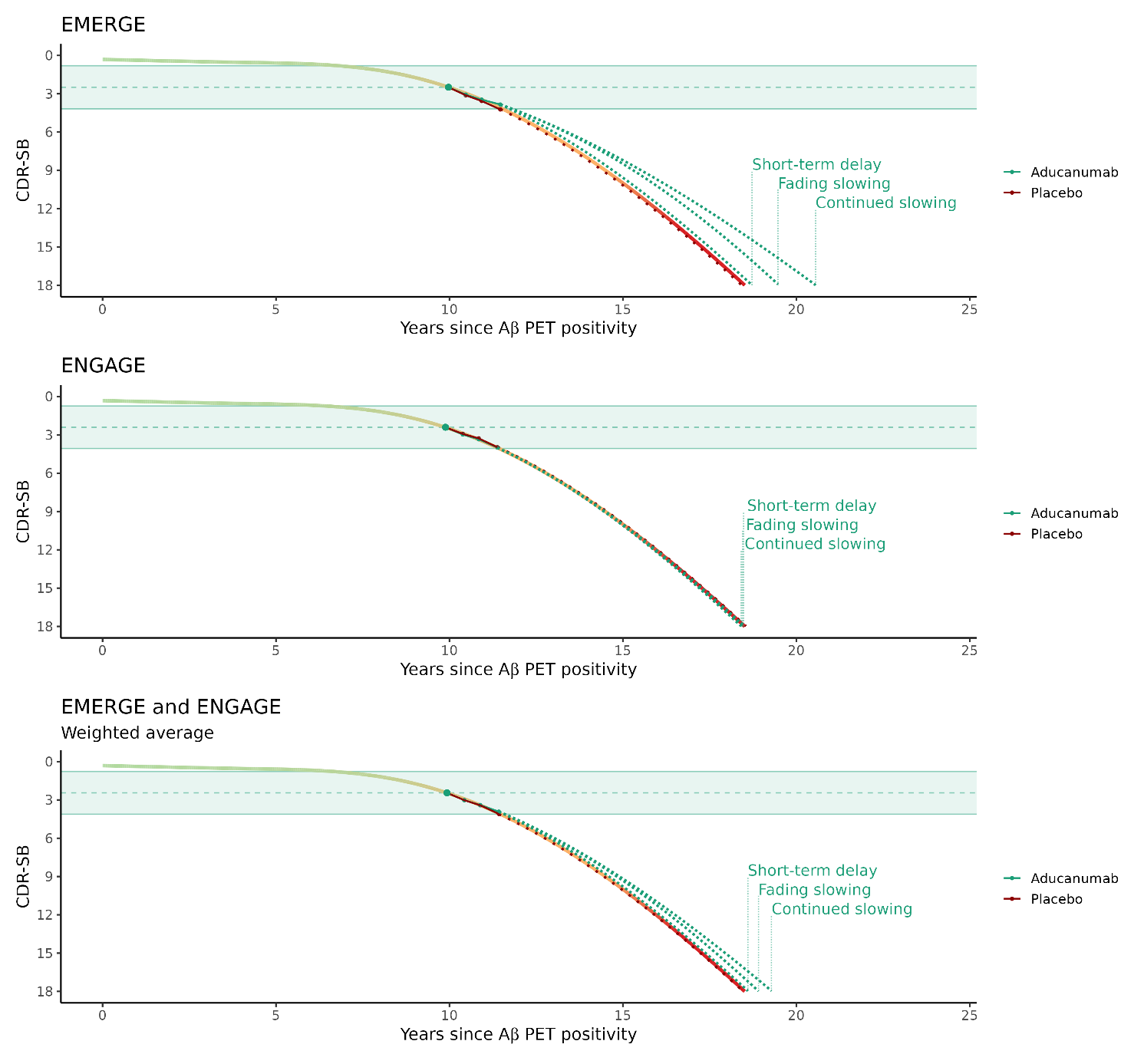


**Figure S5**. EMERGE, ENGAGE, and weighted average trial results mapped to the long-term CDR-SB trajectory with extrapolations (dotted lines). The gradient trajectory represents the estimated natural history trajectory, and the shaded bands represent the 90% prediction intervals for baseline CDR-SB scores in the different populations.


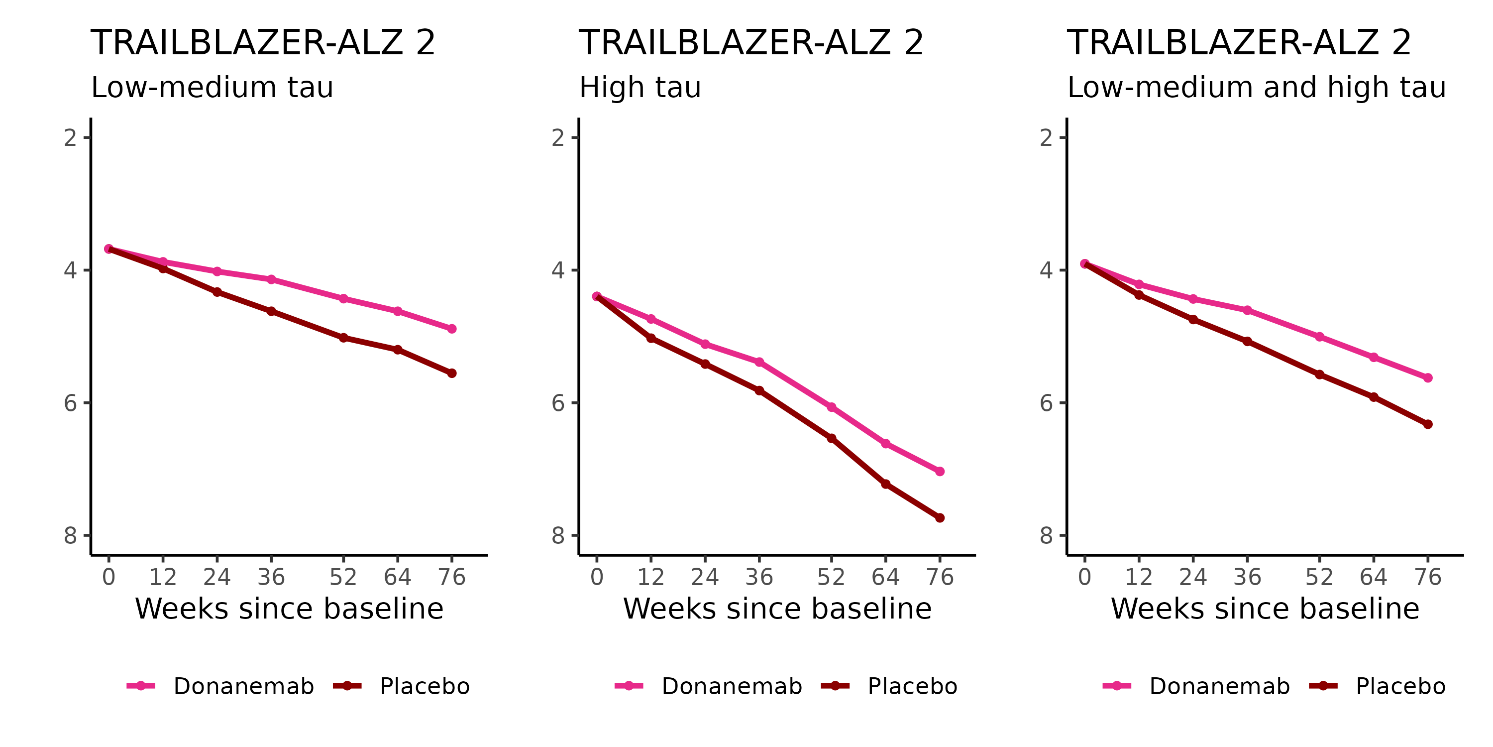


**Figure S6**. Clinical Dementia Rating sum of boxes (CDR-SB) results of the TRAILBLAZER-ALZ 2 trial for the primary low-medium tau population, the high tau population, and the combined low-medium and high tau population. Results are based on previously reported results of change from baseline in CDR-SB analyzed using the mixed model for repeated measures with the reported average baseline score added to the results to bring them to the CDR-SB scale.


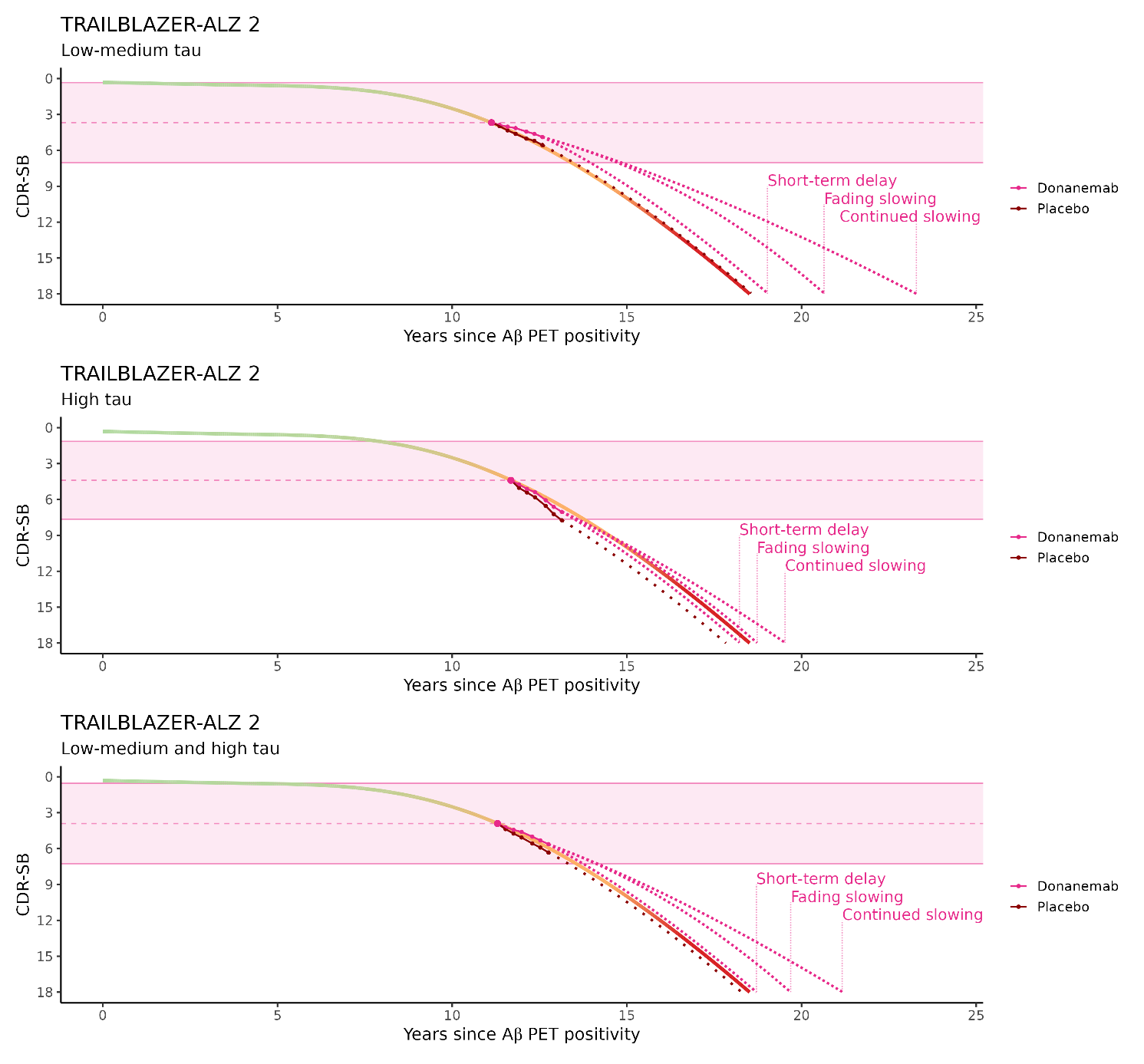


**Figure S7.** TRAILBLAZER-ALZ 2 results of the primary low-medium tau population, the high tau population, and the combined low-medium and high tau population mapped to the long-term CDR-SB trajectory with extrapolations (dotted lines). The gradient trajectory represents the estimated natural history trajectory, and the shaded bands represent the 90% prediction intervals for baseline CDR-SB scores in the different populations.

**Table S3.** Extrapolated treatment effect on CDR-SB after visit 3, 4, and 5 years for the three different scenarios across trials. TRAILBLAZER-ALZ results refers to the primary low-medium tau population.

**a. Enduring short-term delay**

| Trial | Treatment | Years after initiation | CDR-SB difference to extrapolated placebo | Time delay (years) | Time saving |
| --- | --- | --- | --- | --- | --- |
| EMERGE | Aducanumab | 3 | -0.48 | 0.30 | 10% |
| ENGAGE | Aducanumab | 3 | 0.07 | -0.02 | -1% |
| CLARITY AD | Lecanemab | 3 | -0.55 | 0.47 | 16% |
| GRADUATE I | Gantenerumab | 3 | -0.32 | 0.12 | 4% |
| GRADUATE II | Gantenerumab | 3 | -0.23 | 0.21 | 7% |
| TRAILBLAZER-ALZ 2 | Donanemab | 3 | -0.84 | 0.58 | 19% |
| EMERGE | Aducanumab | 4 | -0.54 | 0.30 | 8% |
| ENGAGE | Aducanumab | 4 | 0.08 | -0.02 | -1% |
| CLARITY AD | Lecanemab | 4 | -0.61 | 0.47 | 12% |
| GRADUATE I | Gantenerumab | 4 | -0.35 | 0.12 | 3% |
| GRADUATE II | Gantenerumab | 4 | -0.25 | 0.21 | 5% |
| TRAILBLAZER-ALZ 2 | Donanemab | 4 | -0.93 | 0.58 | 14% |
| EMERGE | Aducanumab | 5 | -0.60 | 0.30 | 6% |
| ENGAGE | Aducanumab | 5 | 0.09 | -0.02 | -0% |
| CLARITY AD | Lecanemab | 5 | -0.67 | 0.47 | 9% |
| GRADUATE I | Gantenerumab | 5 | -0.38 | 0.12 | 2% |
| GRADUATE II | Gantenerumab | 5 | -0.28 | 0.21 | 4% |
| TRAILBLAZER-ALZ 2 | Donanemab | 5 | -1.01 | 0.58 | 12% |

**b. Fading stage-dependent slowing**

| Trial | Treatment | Years after initiation | CDR-SB difference to extrapolated placebo | Time delay (years) | Time saving |
| --- | --- | --- | --- | --- | --- |
| EMERGE | Aducanumab | 3 | -0.91 | 0.57 | 19% |
| ENGAGE | Aducanumab | 3 | 0.10 | -0.04 | -1% |
| CLARITY AD | Lecanemab | 3 | -1.25 | 0.87 | 29% |
| GRADUATE I | Gantenerumab | 3 | -0.39 | 0.15 | 5% |
| GRADUATE II | Gantenerumab | 3 | -0.36 | 0.28 | 9% |
| TRAILBLAZER-ALZ 2 | Donanemab | 3 | -1.83 | 1.16 | 37% |
| EMERGE | Aducanumab | 4 | -1.30 | 0.72 | 18% |
| ENGAGE | Aducanumab | 4 | 0.13 | -0.05 | -1% |
| CLARITY AD | Lecanemab | 4 | -1.84 | 1.09 | 27% |
| GRADUATE I | Gantenerumab | 4 | -0.50 | 0.19 | 5% |
| GRADUATE II | Gantenerumab | 4 | -0.54 | 0.35 | 9% |
| TRAILBLAZER-ALZ 2 | Donanemab | 4 | -2.60 | 1.55 | 35% |
| EMERGE | Aducanumab | 5 | -1.67 | 0.84 | 17% |
| ENGAGE | Aducanumab | 5 | 0.16 | -0.06 | -1% |
| CLARITY AD | Lecanemab | 5 | -2.40 | 1.27 | 25% |
| GRADUATE I | Gantenerumab | 5 | -0.60 | 0.22 | 4% |
| GRADUATE II | Gantenerumab | 5 | -0.70 | 0.40 | 8% |
| TRAILBLAZER-ALZ 2 | Donanemab | 5 | -3.33 | 1.93 | 33% |

**c. Continued stage-independent slowing**

| Trial | Treatment | Years after initiation | CDR-SB difference to extrapolated placebo | Time delay (years) | Time saving |
| --- | --- | --- | --- | --- | --- |
| EMERGE | Aducanumab | 3 | -0.95 | 0.61 | 20% |
| ENGAGE | Aducanumab | 3 | 0.10 | -0.04 | -1% |
| CLARITY AD | Lecanemab | 3 | -1.28 | 0.92 | 31% |
| GRADUATE I | Gantenerumab | 3 | -0.40 | 0.16 | 5% |
| GRADUATE II | Gantenerumab | 3 | -0.36 | 0.29 | 10% |
| TRAILBLAZER-ALZ 2 | Donanemab | 3 | -1.88 | 1.19 | 40% |
| EMERGE | Aducanumab | 4 | -1.42 | 0.81 | 20% |
| ENGAGE | Aducanumab | 4 | 0.14 | -0.06 | -1% |
| CLARITY AD | Lecanemab | 4 | -1.98 | 1.23 | 31% |
| GRADUATE I | Gantenerumab | 4 | -0.54 | 0.21 | 5% |
| GRADUATE II | Gantenerumab | 4 | -0.59 | 0.38 | 10% |
| TRAILBLAZER-ALZ 2 | Donanemab | 4 | -2.79 | 1.58 | 40% |
| EMERGE | Aducanumab | 5 | -1.94 | 1.01 | 20% |
| ENGAGE | Aducanumab | 5 | 0.18 | -0.07 | -1% |
| CLARITY AD | Lecanemab | 5 | -2.76 | 1.54 | 31% |
| GRADUATE I | Gantenerumab | 5 | -0.70 | 0.26 | 5% |
| GRADUATE II | Gantenerumab | 5 | -0.84 | 0.48 | 10% |
| TRAILBLAZER-ALZ 2 | Donanemab | 5 | -3.81 | 1.98 | 40% |
